# Bridging the Gaps: The Transformative Effect of Coronavirus Disease 2019 (COVID-19) on Preventive Measures Among Medical and Health Sciences College Students in Madinah, Saudi Arabia

**DOI:** 10.7759/cureus.55131

**Published:** 2024-02-28

**Authors:** Ibrahim H Babikir, Abdulaziz M Alamri, Abdulaziz A Alraddadi, Mohesn N Alhajuj, Mohammad M Alnakhle, Hassan A Alhajuj, Mohammed S Alfahal, Mohammed Elmuttalut

**Affiliations:** 1 Department of Microbiology, Al-Rayan National College of Medicine, Madinah, SAU; 2 Department of Microbiology, College of Laboratory Medicine, University of Khartoum, Khartoum, SDN; 3 Medical School, Al-Rayan National College of Medicine, Madinah, SAU; 4 Department of Psychiatry, Al-Rayan National College of Medicine, Madinah, SAU; 5 Department of Community Medicine, Al-Rayan National College of Medicine, Madinah, SAU

**Keywords:** coronavirus, madinah ksa, medical and health sciences students, preventive measures, covid-19

## Abstract

Background

The global impact of coronavirus disease 2019 (COVID-19) has disrupted the activities of medical and health profession education institutions. This study aimed to determine the impact of COVID-19 on medical and health profession education students' knowledge, attitudes, and practices toward preventive measures and their commitment to precautionary measures before, during, and after the pandemic.

Materials and methods

A cross-sectional study was carried out from January to March 2023 using an online, structured, validated questionnaire survey to gather information from medical and health sciences students from three universities, encompassing five colleges in Madinah, Saudi Arabia. The minimum required sample size was estimated using the Epi Info software as 380. The data was analyzed using IBM SPSS Statistics for Windows, Version 20.0 (Released 2011; IBM Corp., Armonk, New York, United States). Statistical tests including Student's t-test, chi-squared test, and analysis of variance (ANOVA) test were applied.

Results

The findings revealed that personal experiences with COVID-19 infection had a significant impact on students' attitudes and commitment to preventive measures (p<0.05). Among the participants, 172 students (45%) reported having contracted COVID-19. Students with clinical exposure showed a higher level of understanding and adherence to preventive measures (248 students, 68%), compared to pre-clinical students (198 students, 52%) (p<0.05). Positive attitudes were observed toward practices such as sneezing etiquette (289 students, 76%) and flu vaccination (314 students, 83%) (p<0.05). However, negative attitudes were observed toward mask-wearing (155 students, 41%) and social distancing (144 students, 38%), particularly among male students (p<0.05).

Conclusion

The study provided valuable insights into the impact of the COVID-19 pandemic on medical and health sciences students' knowledge, attitudes, and practices toward preventive measures and the importance of introducing COVID-19 prevention measures in the pre-clinical phase as well as mental health support to promote positive attitudes and enhance adherence to preventive measures.

## Introduction

The coronavirus disease 2019 (COVID-19) pandemic, which emerged as a global health emergency in December 2019, has had a profound impact on societies worldwide. The rapid spread of the novel coronavirus led the World Health Organization (WHO) to declare it a pandemic in March 2020 [1]. The virus belongs to the family of coronaviruses, named for their crown-like appearance under an electron microscope due to glycoprotein projections [2].

At the beginning of the pandemic, although it had been circulating for some time, only human-to-human transmission has been proven, and little is known about it. The novel virus is a new strain that has not existed in humans before. Chinese officials identified its entity on January 7, 2020; subsequently, it has been related to severe acute respiratory syndrome coronavirus 2 (SARS-CoV-2) [3]. According to WHO, the family of coronavirus is responsible for a wide range of illnesses ranging from the common cold to more serious situations, including severe acute respiratory syndrome (SARS) and Middle East respiratory syndrome (MERS) [4].

The prevalence of this pandemic disrupted the whole world, especially the healthcare systems. Undoubtedly, these situations lead to many safety risks making it difficult for medical staff, the safety of life, and the continuity of education in medical colleges [5]. There are no specific strategies yet that can effectively prevent or treat this disease [6,7]. However, countries worldwide made efforts to prevent the spread by implementing precautionary measures. Complete or partial lockdown was introduced by a few countries to slow the transmission of COVID-19, while other countries tried to enhance the immune system by starting a healthier style of diet depending on specific types of food to fight the infections and to keep people healthy too.

Health sciences college students, as future healthcare professionals, play a crucial role in understanding and implementing preventive measures to mitigate the spread of the virus. Their awareness and adherence to these preventive measures are essential in safeguarding public health and ensuring effective management of the pandemic [8]. In addition, assessing the impact of COVID-19 infection on the awareness of preventive measures among medical and health sciences college students in Madinah is essential to evaluate the effectiveness of current educational approaches [9].

The COVID-19 pandemic has affected not only physical health but also mental well-being. Medical college students may experience increased stress, anxiety, and burnout due to the challenges posed by the pandemic. Understanding their awareness and attitudes toward preventive measures can shed light on potential sources of stress and inform strategies to support their mental well-being, thus ensuring their overall resilience and capacity to provide high-quality healthcare [8,10].

Even under normal circumstances, medical students are known to have a much higher prevalence of anxiety disorders than the general population, especially those who live in the Middle East and Asia [11]. Previously, it was also shown that anxiety levels were higher among medical students during the SARS-CoV-1 outbreak [12]. In terms of disease control, they have also been subject to various techniques [13]. Other students have been hired for hospital-based roles to combat the health system saturation, while some medical schools have banned students from any patient interaction, as advised by the American Association of Medical Colleges, depriving the students of a crucial component of their curriculum [14].

This study aimed to determine the prevalence of COVID-19 among medical and health sciences college students in Madinah, Saudi Arabia, shedding light on the transmission and associated risk factors. The study also sought to evaluate the level of knowledge, attitudes, and practices among medical and health sciences college students regarding the impact of COVID-19 on preventive measures, aiming to identify knowledge gaps and misconceptions for targeted interventions. Additionally, the study endeavors to explore associated factors influencing students' knowledge, attitudes, and practices related to preventive measures, including socio-demographic characteristics and educational background, contributing to the design of tailored interventions for improved adherence among health sciences college students.

The findings of this research will provide valuable insights for educational institutions and policymakers in enhancing preventive medicine awareness among health sciences college students and supporting their mental well-being during and after the COVID-19 pandemic. Additionally, these findings will contribute to recommendations for future preparedness and response efforts, ensuring a more robust and resilient healthcare workforce [15,16].

## Materials and methods

Study design

An observational cross-sectional study was conducted from January to March 2023 utilizing a structured and validated questionnaire. The primary objective was to assess the knowledge, attitudes, and practices of medical and health sciences college students regarding COVID-19 preventive measures before, during, and after the pandemic and associated factors. Furthermore, the study aimed to determine the prevalence of COVID-19 among these students in addition to measuring the psychological impact of the COVID-19 pandemic on students. The research adopted a quantitative design approach, collecting data on the awareness and experiences of medical and health sciences college students regarding preventive measures and their infection status. The term "before COVID-19" refers to the period from the onset of the pandemic in Wuhan until its first occurrence in Saudi Arabia.

Study setting and population

The study was carried out in the medical and health sciences colleges of Al-Rayan National College of Medicine, Taibah University, and Al-Ghad International Colleges in Madinah, Saudi Arabia. Both male and female medical students participated, while those outside Madinah and those unwilling to participate were excluded from the study. The minimum required sample size was estimated using the Epi Info software as 380.

Data collection methods and measurements

Data were collected online through structured, validated (5-point) Likert-scale questionnaires. Each item received a numerical score for quantitative analysis. Participants received the questionnaire as a link. The study focused on two main variables: COVID-19 infection (independent variable) and students' knowledge, attitudes, and practices of preventive measures (dependent variables).

A pilot study was conducted to ensure the questionnaire's clarity, fluency of questions, and accessibility across different devices and operating systems. The pilot study also assessed item variance, reliability, and convergent/discriminant validity, resulting in a coefficient of global reliability of 0.79.

Data management and analysis plan

Data analysis was carried out using IBM SPSS Statistics for Windows, Version 20.0 (Released 2011; IBM Corp., Armonk, New York, United States). Student's t-test was utilized for comparing continuous variables (when normally distributed), the chi-squared test for assessing the association between qualitative variables (proportions), and the analysis of variance (ANOVA) test for comparing variables with three or more categories. A significance level of p<0.05 was considered statistically significant.

Ethical considerations

Approval was obtained from the Medical Research Ethics Review Board of Al-Rayan National College of Medicine (approval number: HA-03-M-122-023). Participants were informed of the research purpose beforehand, and their consent was obtained. The consent process was conducted in the Arabic language, and participants had the autonomy to decide whether to participate. Confidentiality was maintained, and no identifying data such as names, emails, IDs, or phone numbers were collected.

## Results

Demographic data

In this study, a total of 432 subjects were initially enrolled, with 52 individuals (13.6%) classified as non-responders or having incomplete data. A total of 380 participants from three universities encompassing five colleges were included in the study as detailed in Table 1.

**Table 1:** Demographic data and distribution of participants according to university/college and academic level (n=380)

University/college (n=380)	Number of students (N)	Percentage (%)
Taibah University	200	52.8%
Al-Rayan National College of Medicine	152	40.1%
Al-Ghad International Colleges	28	7.1%
Field/level of study (n=380)		
Medicine	250	66%
Laboratory studies	28	7.1%
Pharmacology	29	7.7%
Nursing	63	16.6%
Dentistry	10	2.6%
Total	380	100%
Pre-clinical students (n=182) (47.8%)		
First year	55	14.5%
Second year	50	13%
Third year	77	20.5%
Total	182	48%
Clinical students (n=198) (52.2%)		
Fourth year	66	17.3%
Fifth year	60	15.7%
Sixth year	72	19%
Total	198	52%

Prevalence of COVID-19 infection and the rate of vaccine intake among participants

The study's findings revealed that a majority of participants, specifically 255 individuals (67%), had contracted COVID-19 infection, while 125 participants (33%) remained uninfected. Additionally, a substantial proportion of the study population, comprising 371 participants (97.9%), had received the COVID-19 vaccine, as detailed in Table 2.

**Table 2:** Prevalence of COVID-19 infection and vaccination status among participants from medical and health sciences in Madinah (n=380) COVID-19: coronavirus disease 2019

COVID-19 infection	Year of infection	Number of students N (%)	Number of students N (%)
Yes	2020	70 (18.5%)	255 (67%)
	2021	111 (29.2%)	
	2022	74 (19.3%)	
No	2020-2023	125 (33%)	125 (33%)
Total		380 (100%)	380 (100%)
Doses of COVID-19 vaccine			
Vaccinated	1 dose	31 (8.1%)	372 (97.9%)
	2 doses	116 (30.5%)	
	3 doses	225 (59.3%)	
Not vaccinated	0 dose	8 (2.1%)	8 (2.1%)
Total		380 (100%)	380 (100%)

Knowledge of preventive and control measures

The study findings indicate a notable disparity in knowledge levels between clinical students and pre-clinical students, with the former exhibiting significantly higher knowledge (p<0.05), as revealed in Table 3. Furthermore, the assessment of knowledge related to the Basic Infection Control Skills License (BICSL) Program also demonstrated a significant knowledge gap, favoring clinical students over their pre-clinical counterparts (p<0.05) (Table 3).

**Table 3:** Student's knowledge of preventive medicine and BICSL Program in Madinah (n=380) BICSL: Basic Infection Control Skills License

Student's knowledge of preventive medicine				
Grading	Pre-clinical students N (%)	Clinical students N (%)	Mean±SD	P-value
Don't know	120 (65.9%)	5 (2.5%)	0.74±1.038	0.001
Poor	44 (24.7%)	13 (6.5%)		
Fair	4 (2.2%)	9 (4.5%)		
Good	7 (3.8%)	55 (27.8%)		
Excellent	7 (3.8%)	116 (58.7%)		
Total	182 (100%)	198 (100%)		
Student's knowledge of the BICSL Program				
Grading	Pre-clinical students N (%)	Clinical students N (%)	Mean±SD	P-value
Don't know	146 (80.3%)	0 (0%)	2.26±2.286	0.001
Poor	21 (11.5%)	8 (4%)		
Fair	9 (4.9%)	2 (1%)		
Good	6 (3.3%)	73 (36.8%)		
Excellent	0 (0%)	115 (58.2%)		
Total	182 (100%)	198 (100%)		

Student's attitudes to reduce the spread of COVID-19

The study results revealed that individuals who had contracted COVID-19 exhibited more positive attitudes in comparison to those who remained uninfected. Furthermore, the infected group demonstrated a higher commitment to precautionary measures, as detailed in Table 4.

**Table 4:** Student's attitudes and practices to reduce the spread of COVID-19 in Madinah (n=380) COVID-19: coronavirus disease 2019

Item	Positive feedback N (%)	Negative feedback N (%)	Total N (%)	Mean±SD	P-value
Advising others in COVID-19 vaccination	350 (92.2%)	30 (7.8%)	380 (100%)	1.8±0.270	0.001
Advising others in seasonal flu vaccine	328 (86.3%)	52 (13.7%)	380 (100%)	1.14±0.344	0.001
Advising others in preventive medicine	285 (75.2%)	95 (24.8%)	380 (100%)	1.25±0.434	0.001
Wearing a mask for respiratory problems	230 (60.7%)	150 (39.3%)	380 (100%)	1.39±0.489	0.021
Sneezing contact	227 (59.6%)	153 (40.4%)	380 (100%)	1.40±0.491	0.021
Avoiding crowded places	225 (59.4%)	155 (40.6%)	380 (100%)	1.41±0.492	0.030

Student's practices to reduce the spread of COVID-19

The study findings indicated that students expressed a notably high willingness to advise others about vaccination against COVID-19, with 350 individuals (92.2%) endorsing this sentiment. Following closely, 328 students (86.3%) conveyed their readiness to counsel others regarding the seasonal influenza vaccine, as depicted in Table 4.

Student's attitudes and practices of preventive measures before and after COVID-19

The findings clearly demonstrated that pre-clinical students exhibited a significantly lower level of knowledge compared to their clinical counterparts, as indicated in Table 5.

**Table 5:** Student's attitudes and practices of preventive measures before and after the COVID-19 pandemic in Madinah (n=380) COVID-19: coronavirus disease 2019

Item 380 (100%)	Scale	Poor N (%)	Not bad N (%)	Fair N (%)	Good N (%)	Excellent N (%)	Mean±SD	P-value
Importance of vaccine	Before	90 (23.7%)	88 (23.2%)	81 (21.4%)	62 (16.4%)	59 (15.5%)	2.77±1.384	0.001
	After	51 (13.5%)	76 (20.1%)	60 (15.8%)	84 (22.2%)	109 (28.7%)	3.33±1.416	
Wearing of a facemask (among females)	Before	108 (28.5%)	97 (25.6%)	69 (18.2%)	65 (17.2%)	41 (10.8%)	2.56±1.345	0.002
	After	38 (10%)	57 (15%)	86 (22.7%)	91 (24%)	108 (28.4%)	3.46±1.312	
Washing hands	Before	73 (19.3%)	112 (29.6%)	67 (17.7%)	79 (20.8%)	49 (12.9%)	2.79±1.321	0.021
	After	49 (12.9%)	63 (16.6%)	86 (22.7%)	87 (23%)	95 (25%)	3.31±1.350	
Social distancing	Before	73 (19.2%)	110 (28.9%)	83 (21.8%)	75 (19.7%)	39 (10.3%)	2.73±1.264	0.693
	After	70 (18.4%)	80 (21.1%)	80 (21.1%)	101 (26.6%)	49 (12.9%)	2.94±1.315	
Cough etiquette	Before	91 (24%)	102 (26.9%)	72 (19%)	79 (20.8%)	36 (9.5%)	2.65±1.302	0.0311
	After	50 (13.2%)	72 (18.9%)	76 (20%)	93 (24.5%)	89 (23.4.2%)	3.26±1.354	

The impact of COVID-19 on attitudes before, during, and after the pandemic

The majority of participants (n=380) exhibited positive attitudes toward recommending COVID-19 vaccination, with a significant p-value of 0.001 (Table 6). Notably, the study revealed a noteworthy shift in participants' mental well-being from before to during the COVID-19 period. Prior to the pandemic, a mere seven participants (1.8%) reported feeling depressed, a markedly lower figure compared to the 29 (7.6%) experiencing anxiety and 19 (5%) feeling nervous. However, during the COVID-19 period, these numbers surged significantly, with 239 participants (62.9%) grappling with anxiety, 245 (64.5%) experiencing nervousness, and 201 (52.9%) feeling depressed. It's noteworthy that a considerable proportion of participants, specifically 172 (45.3%), reported feeling depressed, while 112 (29.5%) experienced anxiety, and 116 (30.5%) reported feelings of nervousness (Table 6).

**Table 6:** Student's attitudes and practices toward COVID-19 before, during, and after the pandemic in Madinah (n=380) COVID-19: coronavirus disease 2019

Item N (%)	Before COVID-19 N (%)	During COVID-19 N (%)	After COVID-19 N (%)	Mean±SD	P-value
Advice for pandemic vaccine	29 (7.6%)	250 (65.8%)	101 (26.6%)	2.19±0.554	0.001
Advice for seasonal flu vaccine	55 (14.5%)	216 (56.8%)	109 (28.7%)	2.14±0.642	0.026
Advice for immunity-boosting foods	28 (7.4%)	253 (65.6%)	99 (26.1%)	2.19±0.548	0.001
Advice to sleep early	27 (7.1%)	265 (69.7%)	88 (23.2%)	2.16±0.527	0.001
Advice for preventive medicine	23 (6.1%)	291 (75.6%)	66 (17.4%)	2.11±0.471	0.030
Handwashing	17 (4.5%)	231 (60.8%)	132 (34.7%)	2.30±0.549	0.001
Wearing of a facemask (among males)	90 (23.7%)	147 (38.7%)	143 (37.6%)	2.14±0.772	0.064
Cough and sneezing etiquette	82 (21.6%)	173 (45.5%)	125 (32.9%)	2.11±0.730	0.057
Feeling anxious	29 (7.6%)	239 (62.9%)	112 (29.5%)	2.22±0.569	0.001
Feeling nervous	19 (5%)	245 (64.5%)	116 (30.5%)	2.26±0.539	0.001
Feeling depressed	7 (1.8%)	201 (52.9%)	172 (45.3%)	2.43±0.532	0.001

## Discussion

The COVID-19 pandemic had a significant impact on various aspects of life. Our study aimed to evaluate the effect of COVID-19 infection on the knowledge, attitudes, and practices of medical and health sciences students toward preventive measures. Of the 380 participants, 255 (67%) had experienced a COVID-19 infection since 2020, while the remaining 125 (33%) had not. 

The study highlighted the impact of personal experiences on students' knowledge, attitudes, and practices toward preventive measures. For instance, students who had been infected with COVID-19 showed more positive attitudes and higher commitment to precautionary measures compared to those who had not been infected. A similar finding was reported in a study conducted by Johnson et al. (2022) which revealed that individuals with prior personal experiences of infectious diseases exhibited higher knowledge, positive attitudes, and adherence to preventive measures during the COVID-19 pandemic [17]. This finding underscores the importance of personal experiences in shaping individuals' perceptions and behaviors. These students can serve as role models and advocates for preventive measures, encouraging their peers and the wider community to adopt similar attitudes and practices.

Our findings also indicated that the majority of participants displayed positive attitudes toward accepting vaccines and dealing with COVID-19. This finding is consistent with previous studies carried out in Saudi Arabia [18]. Vaccinated medical students also tend to have positive attitudes toward vaccines and encourage others to get vaccinated. This study revealed that only a small proportion (2.1%) of the participating students hesitated about the vaccine. This low hesitancy rate is allying with previous research study findings [19,20]. This can be attributed to their educational background, exposure to scientific information, the impact of the COVID-19 pandemic, and their trust in the Saudi Ministry of Health and Saudi authorities.

It is important to note the study findings revealed significant differences in knowledge, attitudes, and practices toward preventive measures and infection control between pre-clinical and clinical students, suggesting that pre-clinical students had lower knowledge compared to their clinical counterparts. This result was supported by a similar finding in a study carried out in 2020 by Chen and colleagues which indicated that clinical medical students displayed higher knowledge, more positive attitudes, and better adherence to preventive measures compared to pre-clinical students, emphasizing the impact of clinical exposure on infection control practices [21]. This difference suggests that clinical exposure and hands-on experience may contribute to a better understanding of preventive measures. To bridge this knowledge gap, it is crucial to integrate preventive medicine and infection control training early in the medical curriculum to ensure that all students receive comprehensive education on these topics.

Most participants expressed positive attitudes toward practices such as sneezing etiquette, getting the seasonal flu vaccine, avoiding crowded places, and advising others on preventive measures and vaccination. Statistical analysis confirmed the significance of these attitudes. However, there were negative attitudes and practices toward preventive measures, such as wearing masks and practicing social distancing, particularly among male students.

The study results also showed a significant difference between male and female participants regarding mask-wearing. Face masking was found to be beneficial in reducing the transmission of COVID-19, with a high compliance rate among both females (90%) and males (76.6%). This finding is consistent with previous research studies that had shown gender differences in adherence to preventive measures during the pandemic [19,22]. This may be attributed to the exciting cultural effect that encourages the female to cover their face.

Positive attitudes were observed toward other preventive measures such as early sleep and consuming immunity-boosting foods. Most participants agreed on the importance of using immunity-boosting foods before, during, and after COVID-19. Previous research study findings have also highlighted the significant impact of dietary and herbal behaviors on preventive measures [22].

Prior to COVID-19, the incidence of depression among students was relatively low at 1.8%, while anxiety and nervousness exhibited higher rates at 7.6% and 5%, respectively. These findings suggest that students already experienced higher levels of anxiety and nervousness even before the pandemic and depression was less prevalent among students prior to the COVID-19 outbreak. During the COVID-19 period, there was a significant increase in the rates of anxiety, nervousness, and depression among students. The percentage of students feeling anxious was 62.9%, feeling nervous was 64.5%, and feeling depressed was 52.9%. These results indicate a substantial surge in anxiety and nervousness symptoms among students during the pandemic and highlighted the COVID-19 pandemic as a risk factor for mental health problems. Numerous previous research studies have explored the factors associated with the emergence of psychological problems such as stress, anxiety, and depression during the COVID-19 pandemic [23,24].

Furthermore, the incidence of depression during COVID-19 was higher compared to the rates of anxiety and nervousness. Specifically, depression was reported by 45.3% of students, while anxiety was reported by 29.5% and nervousness by 30.5%. This suggests that students might have been more prone to experiencing depressive symptoms compared to anxiety and nervousness during the pandemic. This finding is consistent with the result of a systematic review study conducted by Dong and colleagues on psychological problems in individuals with COVID-19 which revealed high proportions of patients experiencing anxiety, depression, and post-traumatic stress disorder [25]. These studies further support the prevalence and impact of psychological issues during the pandemic.

This study has several strengths, including its longitudinal analysis tracking changes in students' knowledge, attitudes, and practices toward COVID-19 over time and adequate sample size. However, limitations include reliance on self-reported measures, potential cross-cultural variations, and the absence of a control group.

## Conclusions

This study highlighted the impact of the COVID-19 pandemic on medical and health sciences students' knowledge, attitudes, and practices toward preventive measures. Notably, clinical students exhibited greater knowledge and more positive attitudes compared to pre-clinical students, who demonstrated significantly lower knowledge and more negative attitudes, particularly toward COVID-19 precautionary measures. Negative attitudes and practices, especially related to mask-wearing and social distancing, were evident among pre-clinical students. Factors such as the prolonged duration of the pandemic, lockdowns, and university closures have contributed to increased complications for students. The study recommends the urgent implementation of educational curricula and training courses in preventive measures and infection control, especially targeting pre-clinical students. Additionally, there is a call for more scientific studies addressing the impact of COVID-19 on educational aspects, mental health, and the psychological and social well-being of medical students. Furthermore, the provision of mental health and psychosocial support during pandemics is emphasized.
